# Supplementary material for: Significant stability enhancement in photocatalytic CO2 reduction via flow-driven strategies
Source: Nat Commun. 2026 Mar 18;17:4139. doi: 10.1038/s41467-026-70542-9 (PMC13149638; doi:10.1038/s41467-026-70542-9)
Supplement: Supplementary file 1 — Supplementary Information [file 41467_2026_70542_MOESM1_ESM.pdf]

Supplementary Materials for

Significant Stability Enhancement in Photocatalytic CO_2 Reduction via Flow-Driven Strategies

Hyunju Jung[1,2,3], Hyo Sang Jeon[4], Min Gyu Kim[5], Aqil Jamal[6], Issam Gereige[6], Chansol Kim[7]*, and Hee-Tae Jung[1,2,3]*

Affiliations:

[1]Department of Chemical and Biomolecular Engineering, Korea Advanced Institute of Science and Technology (KAIST), Daejeon, 34141, South Korea.

[2]KAIST-UC Berkeley-Vietnam National University Climate Change Research Center, Daejeon, 34141, South Korea

[3]Saudi Aramco-KAIST CO_2 Management Center, Daejeon, 34141, South Korea

[4]Sustainable Energy Research Division, Korea Institute of Science and Technology (KIST), Seoul, 02792, South Korea.

[5]Beamline Research Division, Pohang Accelerator Laboratory, Pohang University of Science and Technology, Pohang, 37673, South Korea.

[6]Research and Development Center, Saudi Aramco, Dhahran, 31311, Saudi Arabia

[7]Clean Energy Research Center, Korea Institute of Science and Technology (KIST), Seoul, 02792, South Korea

*Corresponding author. Email: chansolkim@kist.re.kr, heetae@kaist.ac.kr

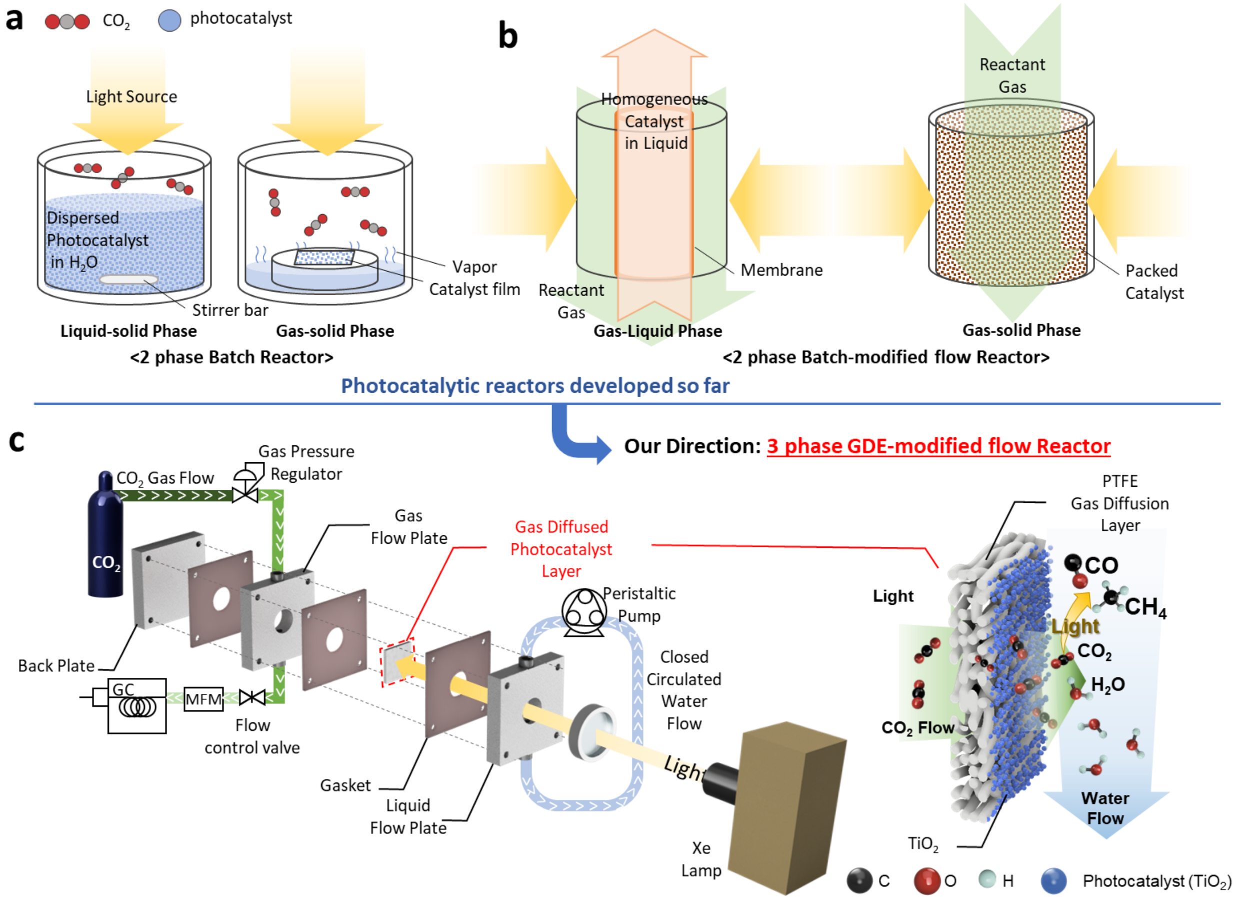

Supplementary Fig. 1 | Photocatalytic Reactor system configuration. **a**, Schematic illustrations of flow-less two-phase batch reactors; **b,** batch-modified flow reactors; and **c,** a gas diffusion electrode (GDE)-modified three-phase flow reactor. (left) Schematic illustration of the continuous flow-enabled photocatalytic reactor showing key components: gas pressure regulator, gas flow plate, mass flow meter (MFM), peristaltic pump, and PTFE membrane with photocatalyst coating. (right) Detailed gas diffused photocatalyst layer highlighting the role of the triple-phase interface in facilitating mass transport of CO_2 and H_2O. Color-coded particles represent C (gray), O (red), H (light blue), and TiO_2 photocatalyst (blue).

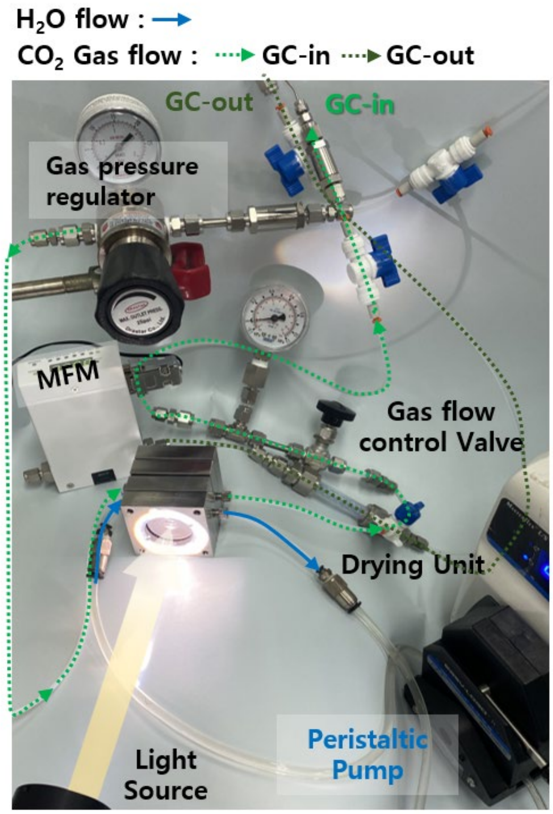

Supplementary Fig. 2 | Photograph of the flow-enabled photocatalytic reactor system

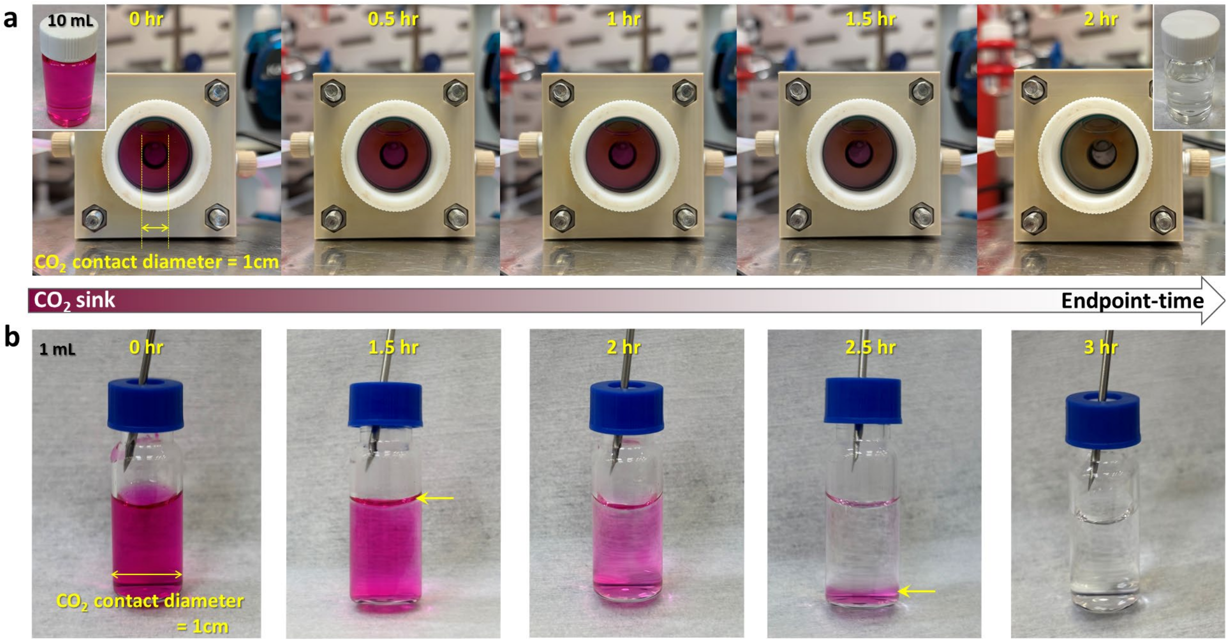

Supplementary Fig. 3 | Time-lapse images of the phenolphthalein decolorization during CO_2 exposure for the same geometric gas-liquid contact area. a, The flow-enabled cell decolorizes uniformly across the interface. Inset: 0.10 M NaOH solution before and after the test. **b**, The flow-less (pressurized gas line in headspace of bottle) cell decolorizes from the gas-liquid interface downward, leaving the bottom region colored longest (yellow arrow), consistent with non-uniform CO_2 delivery and slower intra-liquid mixing in the batch geometry. Contact area = 1cm diameter ≈ 0.785 cm^2, without light irradiation and photocatalyst.

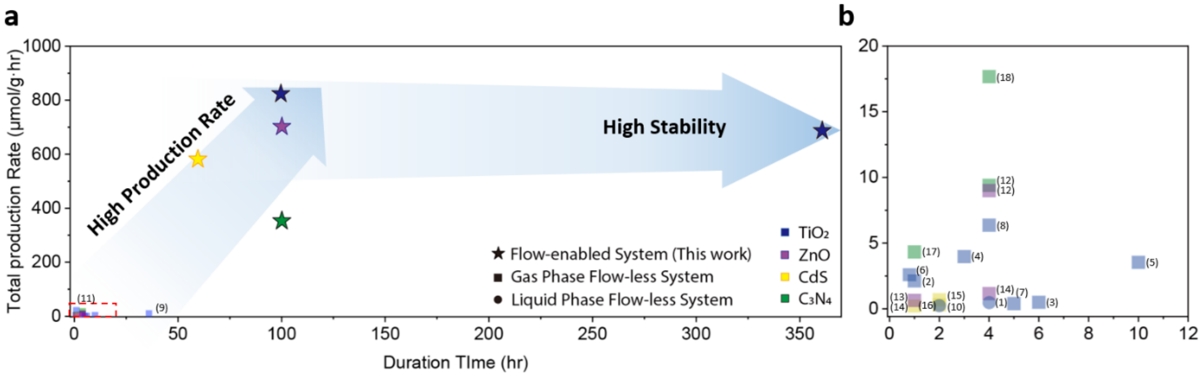

Supplementary Fig. 4 | Performance comparison of various photocatalyst materials. a, Total product formation rates of bare semiconductor photocatalysts (TiO_2, ZnO, CdS, and C_3N_4) as a function of operational duration, showing significantly higher total product formation rates and operational stability in the flow-enabled system compared to conventional gas-phase and liquid-phase flow-less batch systems. **b**, Enlarged view of the region highlighted by the red dashed box in **a**, highlighting the performance of photocatalysts under flow-less batch conditions [1–18].

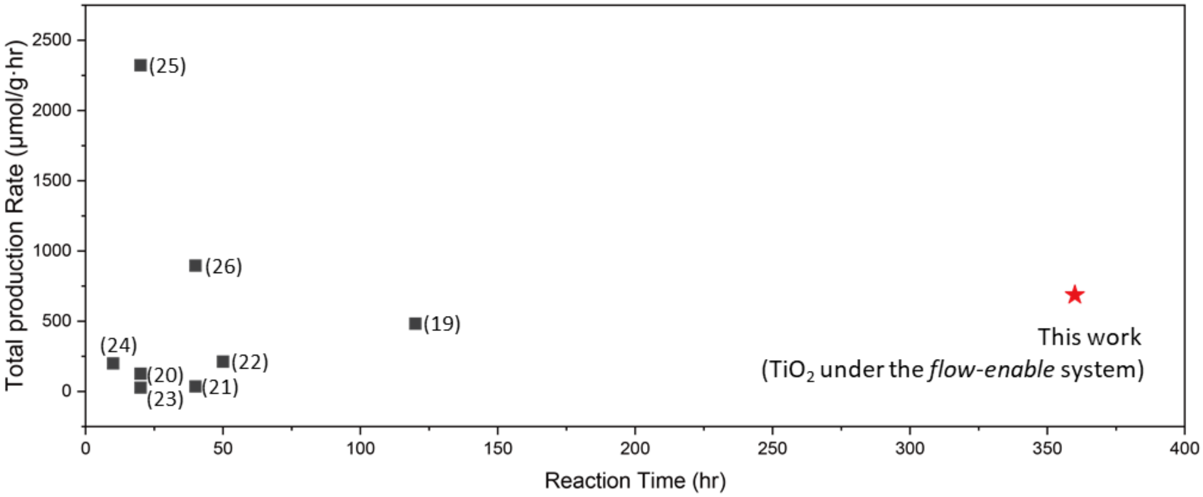

Supplementary Fig. 5 | Comparison of state-of-the-art photocatalytic CO_2 reduction systems. Performance metrics and reaction durations of recently reported photocatalysts [19–26], encompassing various catalyst architectures (e.g., co-catalysts, heterostructures) and reaction conditions (e.g., hole scavengers).

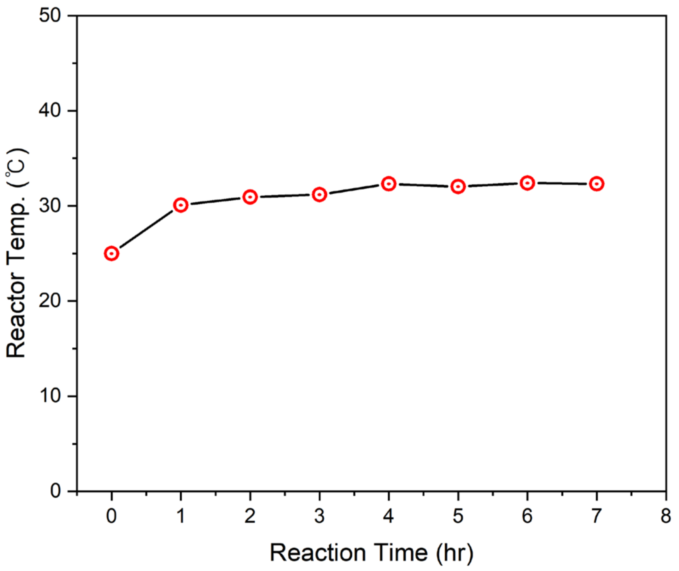

Supplementary Fig. 6 | Internal reactor temperature during flow-enabled operation.

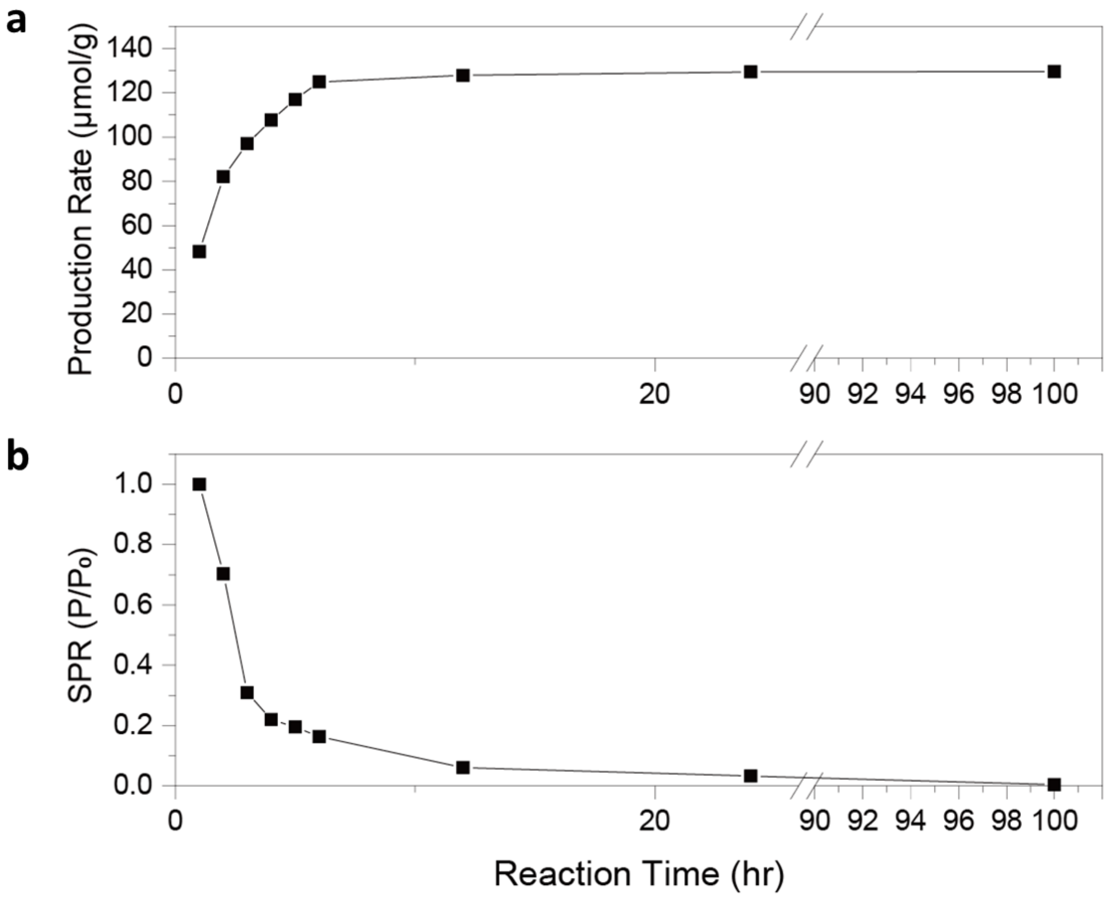

Supplementary Fig. 7 | TiO_2 reaction performance in a flow-less gas-phase batch system. a, Production rate of CO_2 reduction as a function of reaction time. **b**, Corresponding Stability of Photocatalytic Reduction (SPR) value.

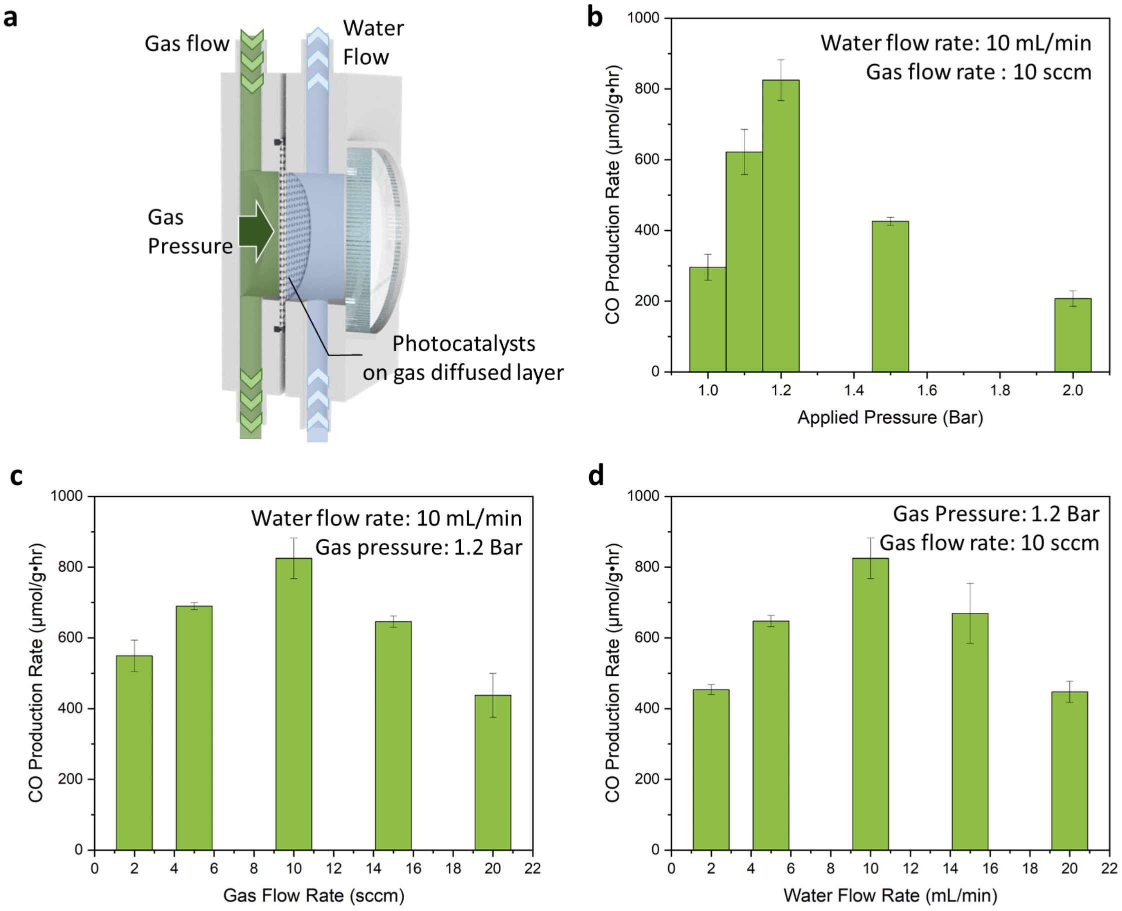

Supplementary Fig. 8 | Effects of various factors on the flow-enabled system. a, Schematic illustration of gas and water flows inside the reactor. **b**, Effect of applied CO_2 gas pressure on the production rate. **c**, Effect of gas flow rate on the production rate. **d**, Effects of the cycled water flow rate on the production rates. Experiments in (b–d) were conducted under 300 mW·cm^{-2} irradiation from a 300 W Xe lamp, with error bars representing standard deviations from at least three identical experiments.

In our flow-enabled system, the formation and stability of the three-phase interface are governed by the interplay between electrolyte flow rate, gas flow rate, and gas pressure, each of which can be independently controlled. The CO_2 gas pressure and gas flow rate are applied on the gas-diffusion side of the catalytic membrane, while the water flow rate corresponds to the liquid side (**Supplementary Fig.8a**). Each parameter has an optimal value: below this threshold, mass transport[27,28] becomes inefficient due to insufficient reactant supply or product removal; above it, the three-phase interface is disrupted—either by electrolyte penetration into the catalyst layer or by reduced residence time that limits effective adsorption and reaction. This behavior is consistent with a transition from a mass-transfer-limited regime to a steady-state regime, confirming that hydrodynamic conditions directly govern catalytic performance.

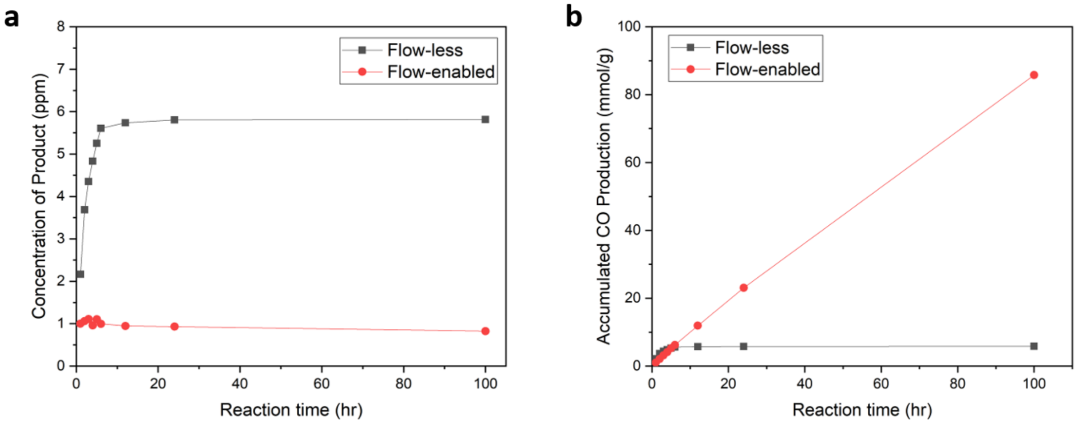

Supplementary Fig. 9 | Comparison of CO production for the flow-less and flow-enabled systems. **a**, time-dependent headspace CO mole fraction (ppm). **b**, accumulated CO production per 1 g of photocatalyst in each reactor.

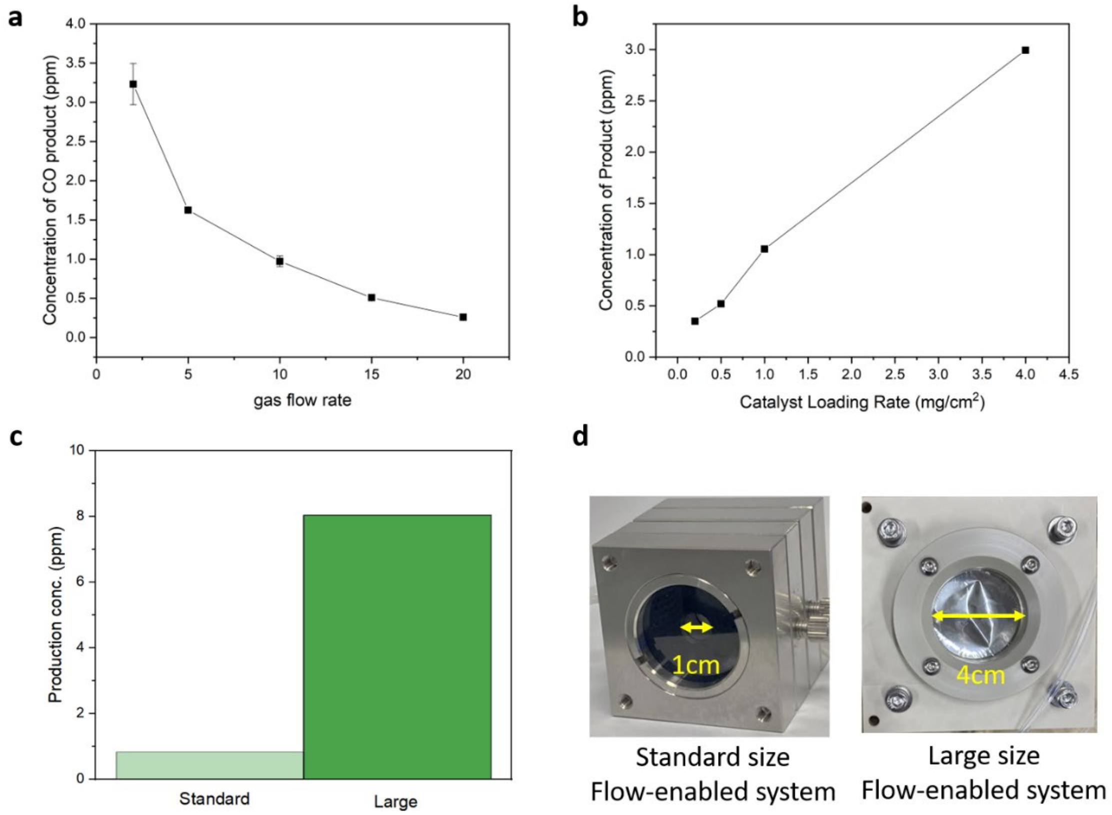

Supplementary Fig. 10 | Tunability of single-pass CO_2 conversion (SPC) in the flow-enabled platform. Outlet concentration as a function of **a**, gas flow rate and **b**, catalyst loading rate. **c**, Effect of reactor illuminated area: comparison between the standard 1 cm diameter aperture and a 4 cm diameter large system. **d**, Photograph of the standard and large size flow-enabled system. All photocatalysts were tested under 300 $mW \cdot cm^{-2}$ illumination with a Xe lamp.

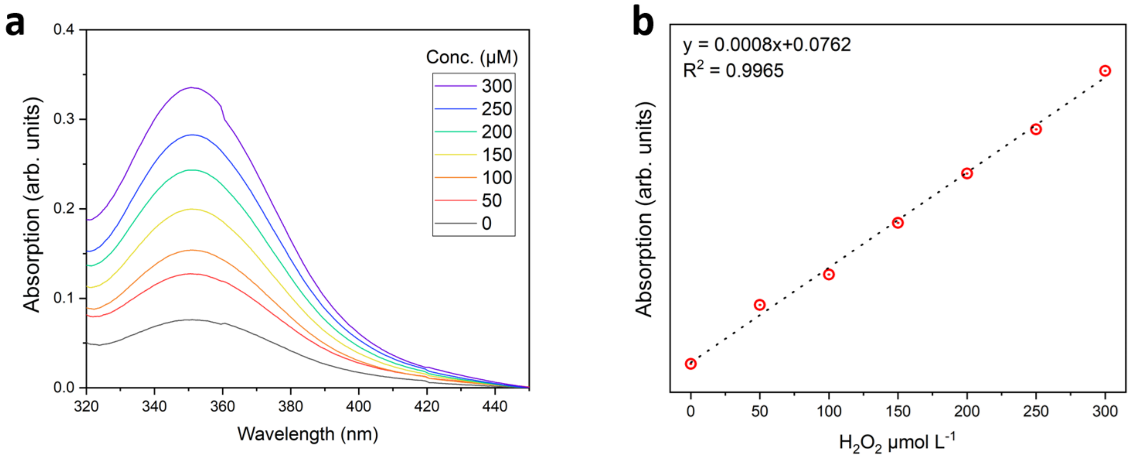

Supplementary Fig. 11 | Quantification of H_2O_2 by iodometry. **a**, UV-vis absorption spectra of standard solutions with increasing H_2O_2 concentration [29]. **b**, Calibration curve at 350 nm (linear fit); H_2O_2 yields in reaction samples were determined from this line.

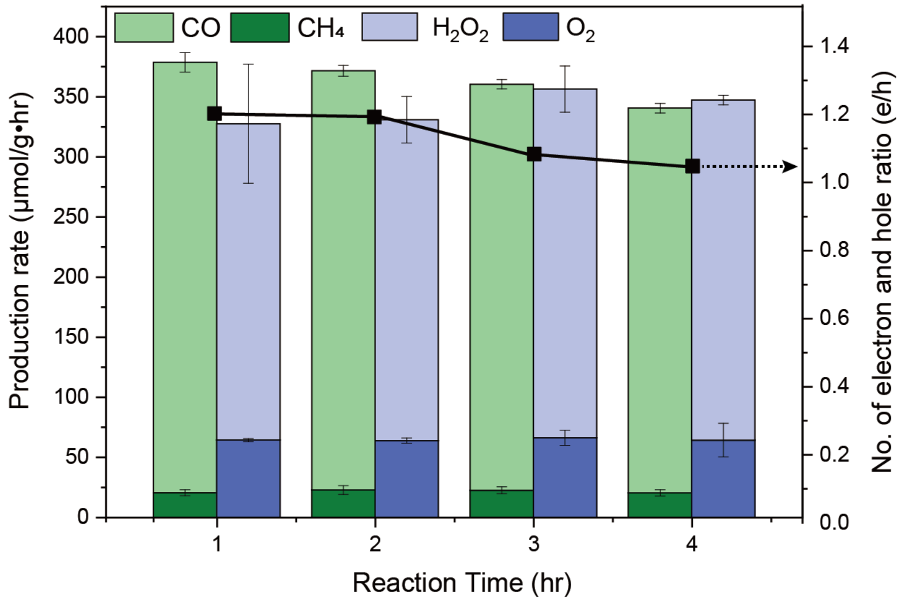

Supplementary Fig. 12 | Comparison of O_2 Production with CO/CH_4 and the e^-/h^+ Ratio under TiO_2 photocatalytic CO_2 reduction under the flow-enabled system. Conditions: TiO_2 1 mg cm^{-2}; CO_2 1.2 bar, 2 sccm; H_2O 10 mL min^{-1} (recirculating); Xe lamp 300 mW cm^{-2}. O_2 by GC–TCD; H_2O_2 by iodometric UV-vis (350 nm). Error bars: two independent experiment. Error bars in **a** represent standard deviations from three replicate experiments. All photocatalysts were tested under 300 mW·cm^{-2} illumination with a Xe lamp.

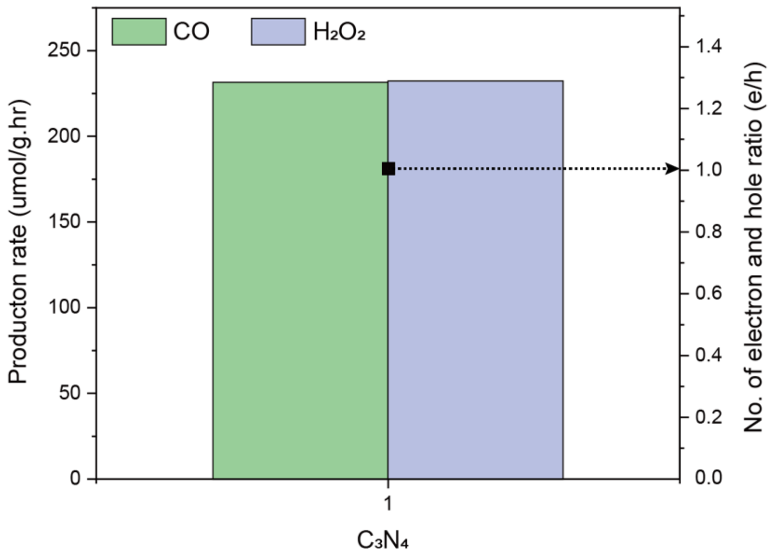

Supplementary Fig. 13 | Comparison of H_2O_2 Production with CO and the e^-/h^+ Ratio under g-C_3N_4 photocatalytic CO_2 reduction under the flow-enabled system under 1 hour visible light irradiation. production rates of H_2O_2 and CO (left) and the e^-/h^+ ratio (right). Conditions: g-C_3N_4 1 mg cm^{-2}; CO_2 1.2 bar, 2 sccm; H_2O 10 mL min^{-1}. All photocatalysts were tested under 300 mW·cm^{-2} illumination with a Xe lamp.

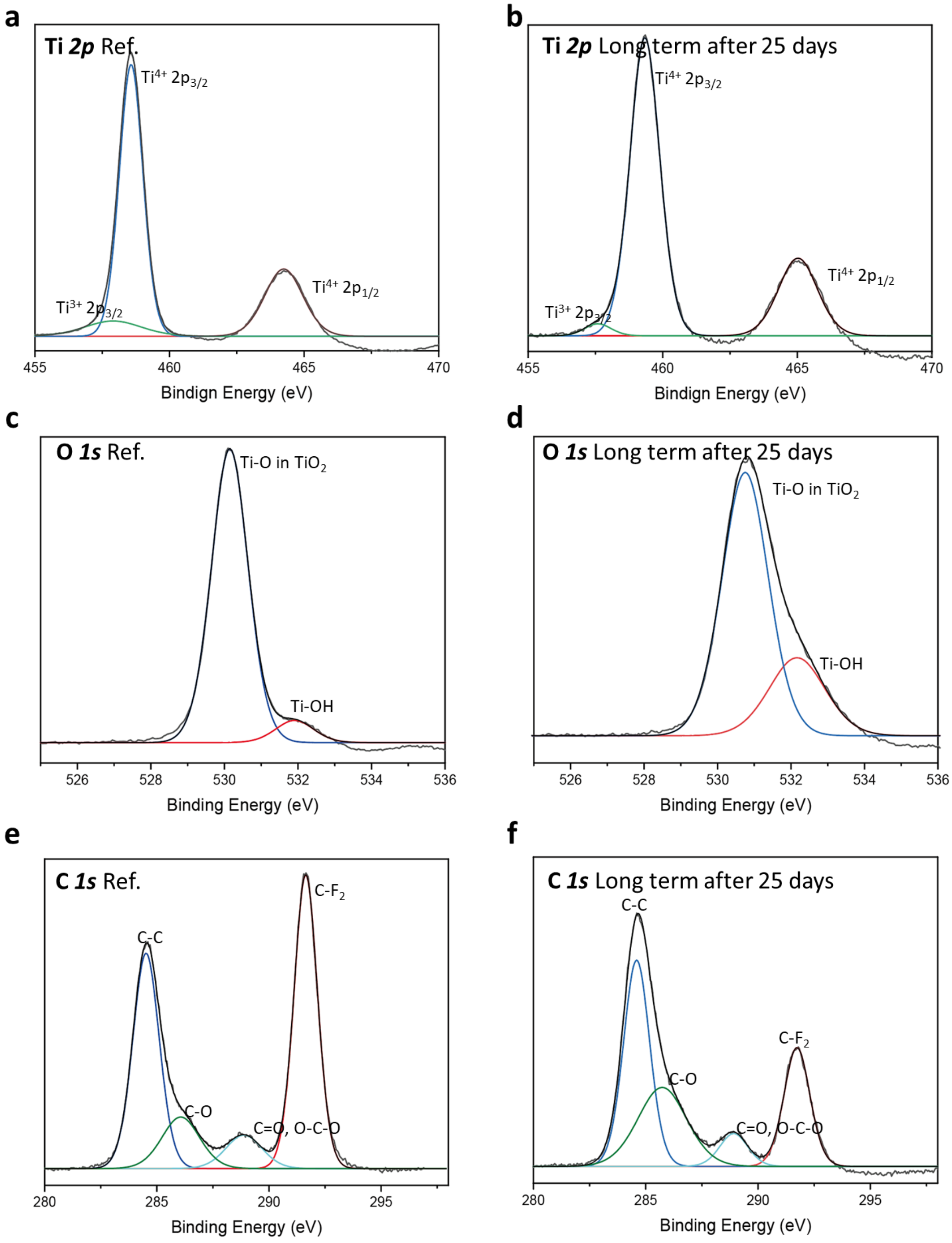

Supplementary Fig. 14 | XPS spectra of the reference photocatalyst layer and the photocatalyst after 25 days of continuous operation. Ti *2p* spectra of **a,** the reference photocatalyst and **b**, the photocatalyst after long-term reaction. O *1s* spectra of **c** the reference and **d** the photocatalyst layer from the flow reactor after 25 days. C *1s* spectra of **e** the reference and **f** the photocatalyst layer from the flow reactor after 25 days

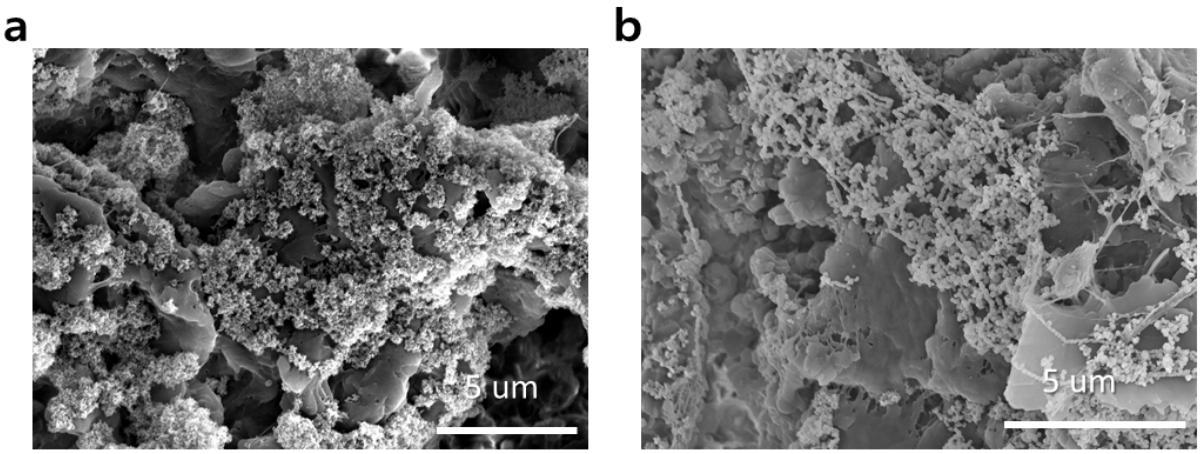

Supplementary Fig. 15 | SEM Image of Gas Diffused Photocatalyst for Super long-term test. **a**, SEM image of the photocatalyst surface before long-term reaction, showing uniform distribution of P25 particles. **b**, SEM image after a 25-day reaction, showing minimal changes in catalyst distribution and surface morphology, indicating the durability and stability of the photocatalyst under prolonged operational conditions.

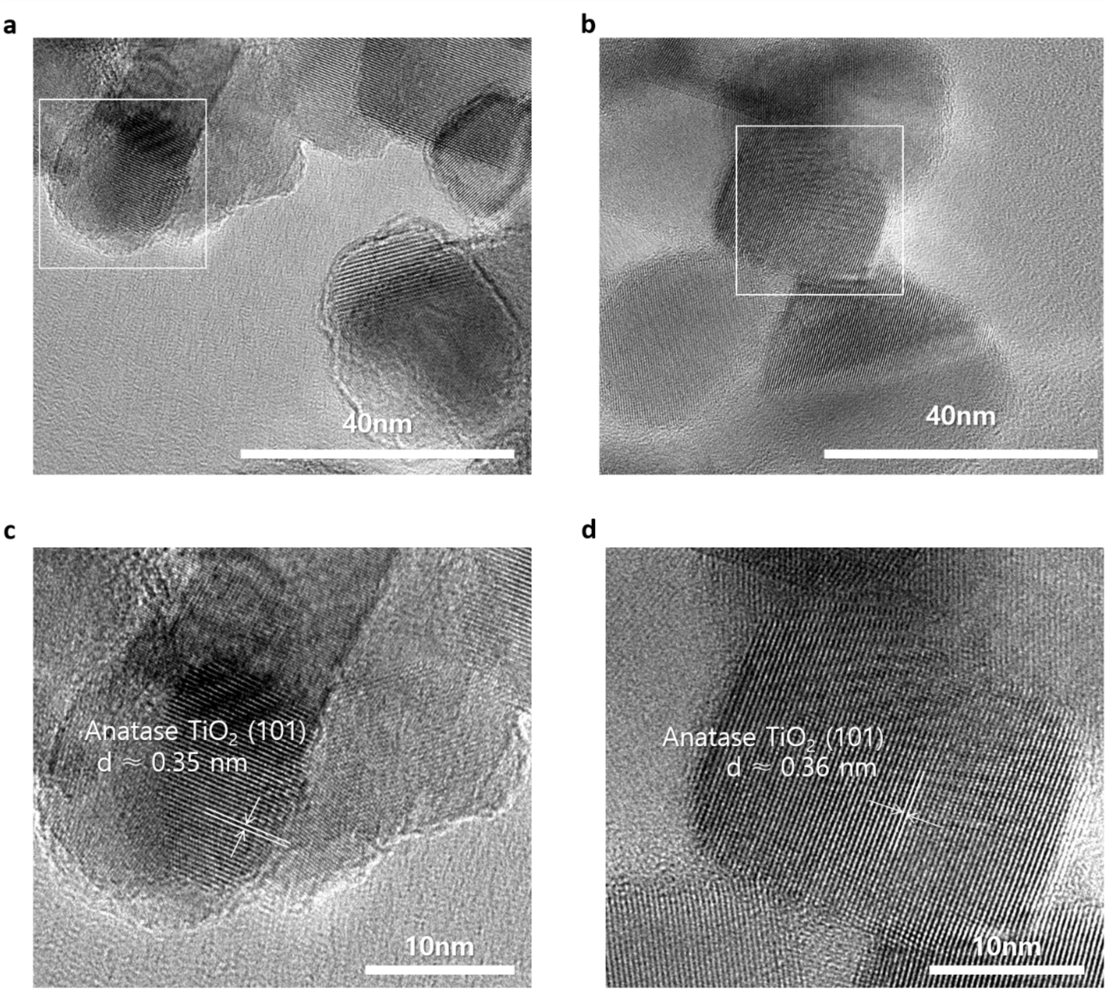

Supplementary Fig. 16 | TEM/HRTEM of TiO_2 before and after long-term flow-enabled operation. Overview of TEM image of a Pristine TiO_2 and **b** the TiO_2 after long-term operation. White boxes mark regions magnified in **c** and **d**, respectively. HRTEM images showing clear lattice fringes with interplanar spacings of d ≈ 0.35–0.36 nm, indexed to anatase (101), indicating preserved crystallinity after reaction.

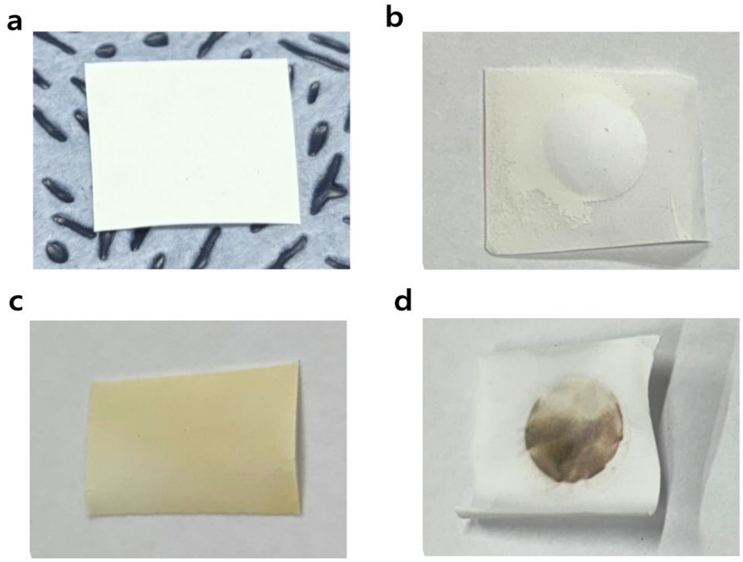

Supplementary Fig. 17 | Visual observations of photocatalyst layers after long-term reactions in flow and batch reactors. a, Reference photocatalyst layer before reaction, showing the pristine color and uniform surface. **b**, The photocatalyst layer from the flow reactor after a 100-hour reaction, exhibiting minimal color changes compared to the reference. **c**, The photocatalyst layer from the batch reactor after a 100-hour reaction, showing noticeable color changes, indicating surface modifications. **d**, The photocatalyst layer from the flow reactor after a 25-day reaction, displaying some color changes, suggesting long-term surface alterations.

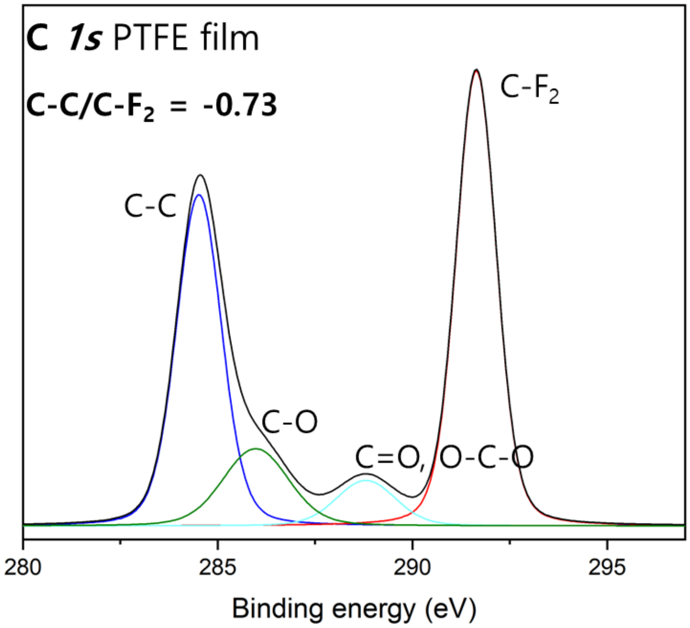

Supplementary Fig. 18 | C *1s* XPS spectrum of bare PTFE film.

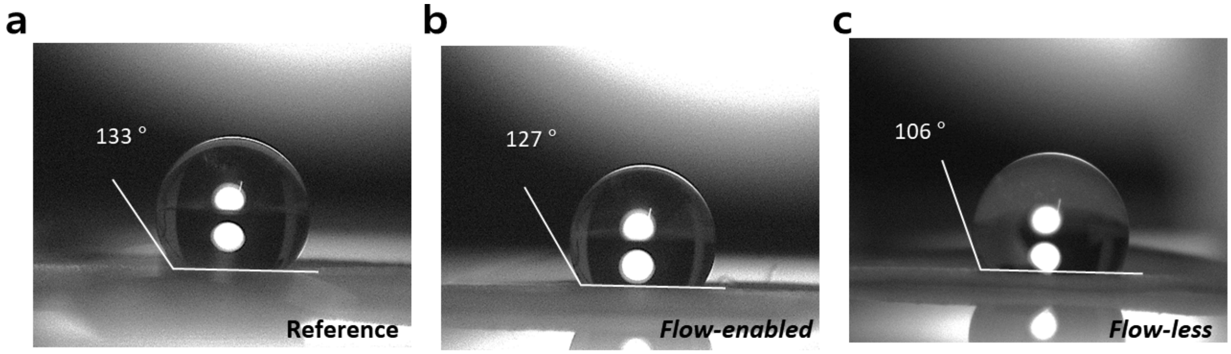

Supplementary Fig. 19 | Contact angle measurements of photocatalyst layers from flow and batch reactors. a, Reference photocatalyst layer before reaction, exhibiting a contact angle of 133°. **b**, The photocatalyst layer from the flow reactor after a 100-hour reaction, showing a contact angle of 127°. **c**, The photocatalyst layer from the batch reactor after a 100-hour reaction, showing a reduced contact angle of 106°, suggesting increased surface hydrophilicity due to accumulated carbon species.

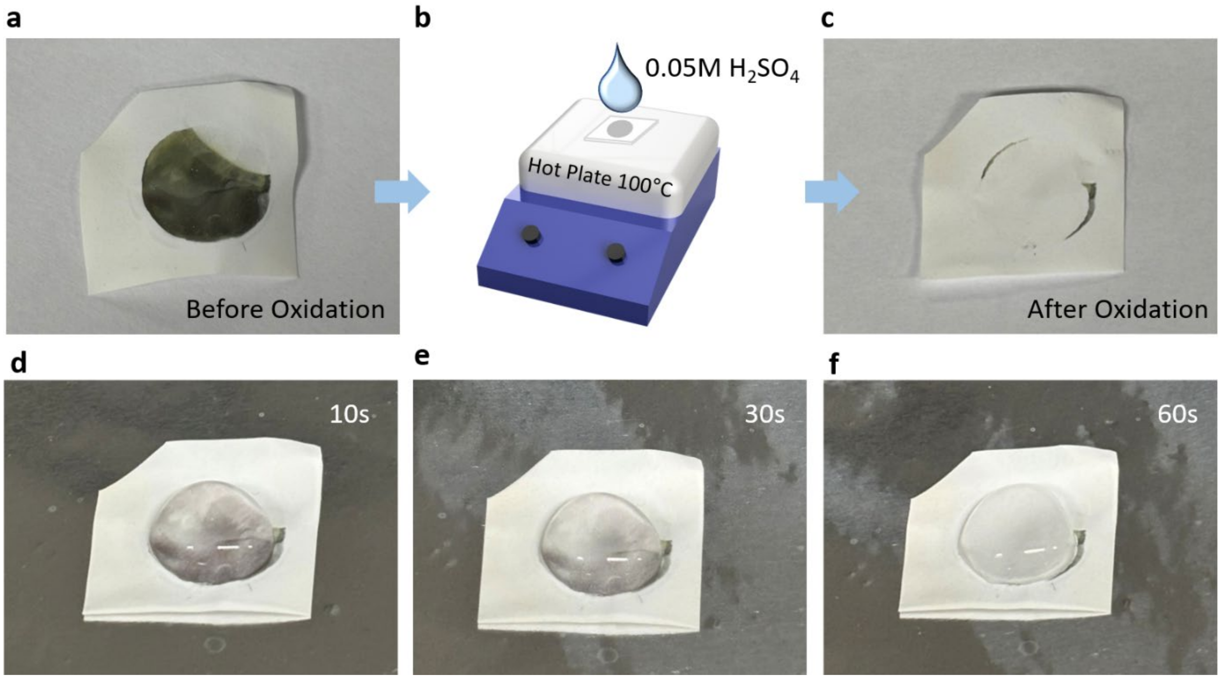

Supplementary Fig. 20 | a, Color change in the thick catalyst layer due to carbon accumulation after deactivation in the flow system. **b**, Oxidation process of the deactivated photocatalyst layer on a 100°C hot plate with a 0.05 M H_2SO_4 droplet. **c**, Recovered photocatalyst layer after the oxidation process, followed by washing with deionized water. **d–f**, Surface changes over time during oxidation: **d**, after 10 s, **e**, after 30 s, and **f**, after 60 s following the addition of sulfuric acid.

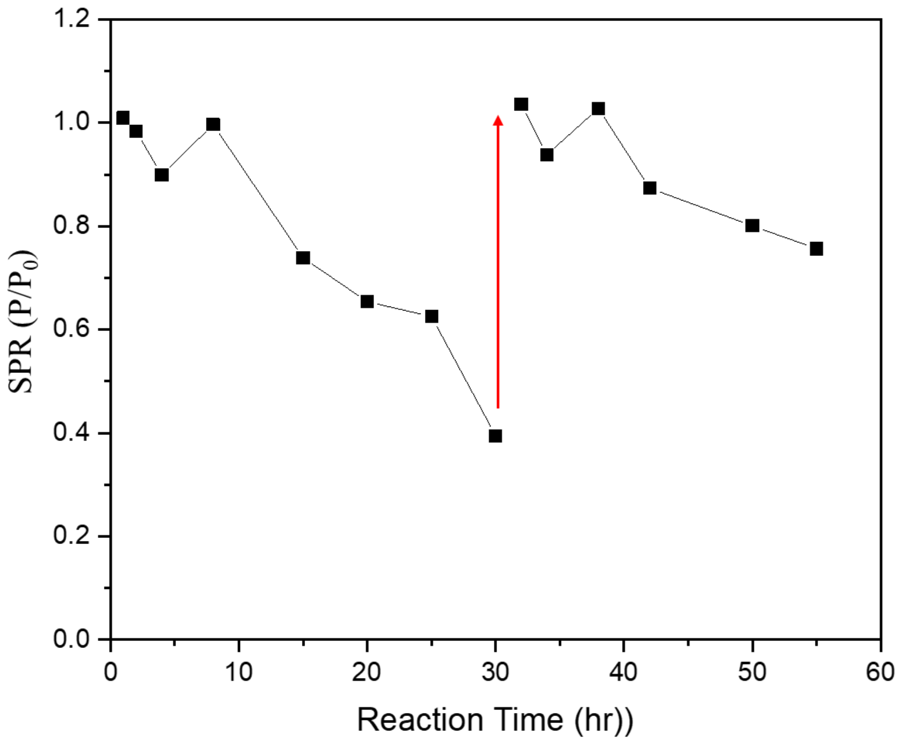

Supplementary Fig. 21 | Recovery of the initial production rate before and after the photocatalyst surface cleaning process.

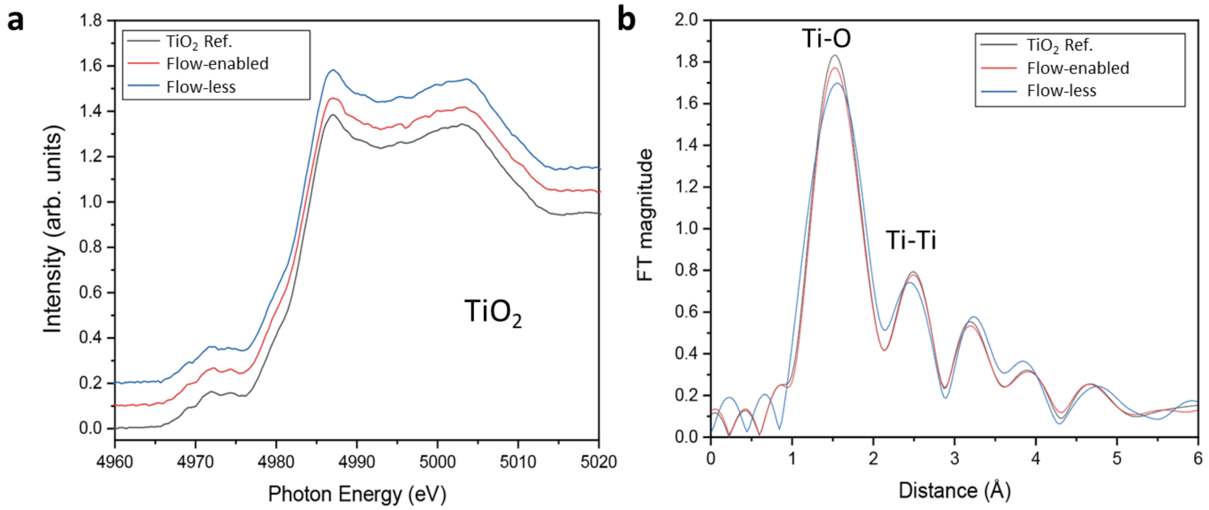

Supplementary Fig. 22 | Structural analysis of TiO_2 photocatalyst after 100hr long-term reactions. **a**, Ti K-edge XANES spectra for bare TiO_2 samples from reference, flow, and batch reactors, and **b**, itsFourier transform of Ti K-edge XANES spectra.

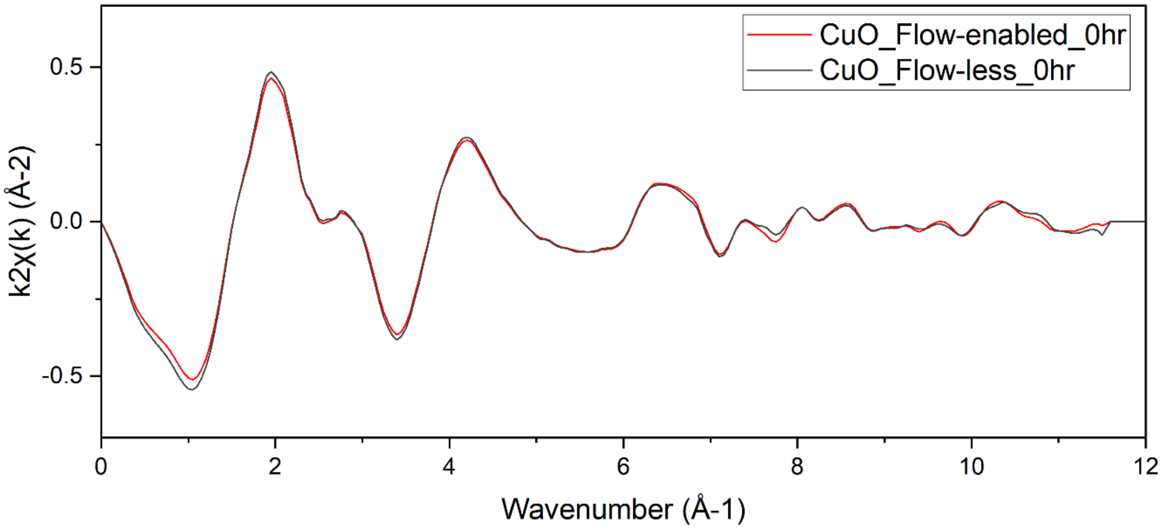

Supplementary Fig. 23 | Cu K-edge XANES k-space of CuO from flow-enabled and flow-less system before reaction

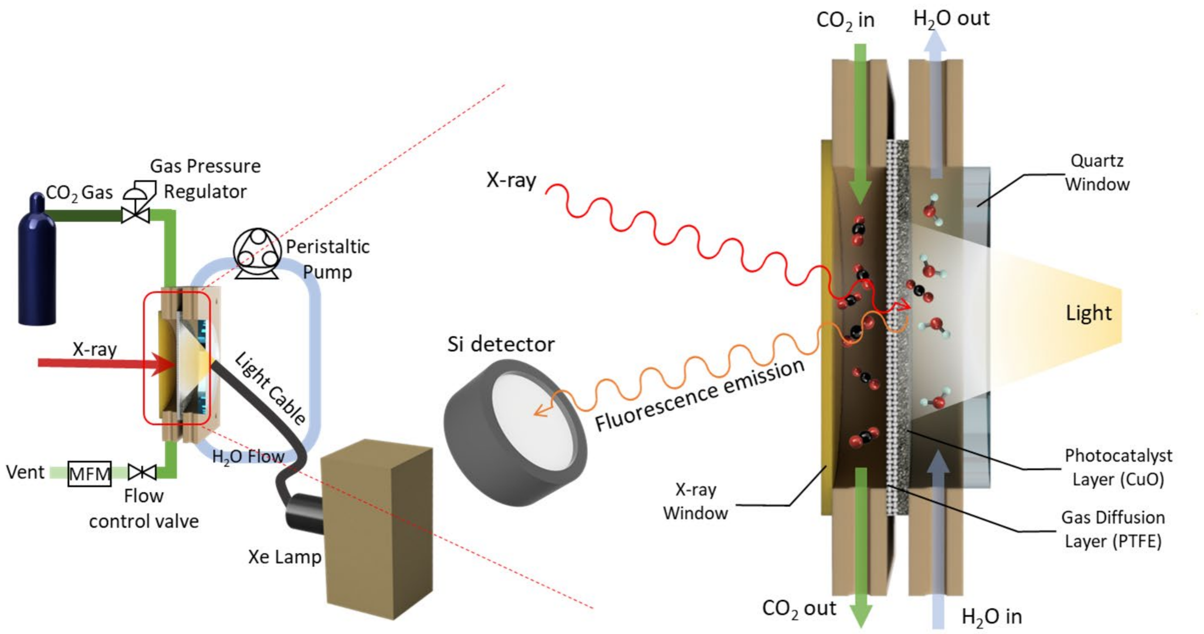

Supplementary Fig. 24 | Schematic of the in-situ synchrotron-compatible continuous flow-enabled system setup, showing the optical fiber delivering light from a Xenon lamp and X-rays passing through a PTFE window to the photocatalyst layer, with fluorescence signals detected by a Si detector

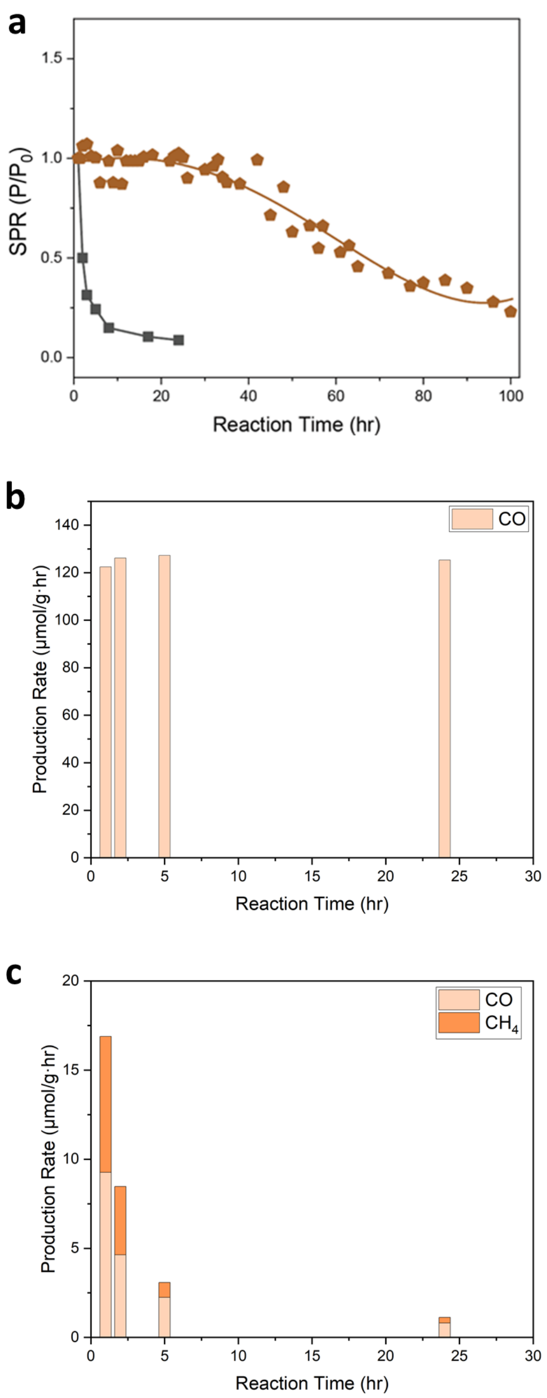

Supplementary Fig. 25 | Performance of CuO under flow-enabled and batch operation. a, Stability over a 100 h reaction. **b**, Production rate of CuO in flow-enabled system and **c**, in flow-less system at 1, 2, 5, 24 h. Reaction conditions: Xe lamp 100 mW cm^{-2}; CO_2 10 sccm; H_2O 10 mL min^{-1}.

Supplementary Table 1 | The performance comparison of our work with semiconductor-based photocatalysts without co-catalysts and hole scavenger for CO_2 reduction.

Photocatalyst[a]	Reaction Medium	Light Source	Production Rate[b] (μmol/g•hr)	Stability[c] (hr)	Reaction system[d]	Ref.
TiO_2 (P25)	CO_2 and H_2O flow	300W Xe Lamp	825	100	Continuous flow reactor	This work
TiO_2 (P25)	CO_2 and H_2O flow	300W Xe Lamp	686	360	Continuous flow reactor	This work
ZnO	CO_2 and H_2O flow	300W Xe Lamp	698	100	Continuous flow reactor	This work
CdS	CO_2 and H_2O flow	300W Xe Lamp with visible filter (> 420nm)	577	100	Continuous flow reactor	This work
C_3N_4	CO_2 and H_2O flow	300W Xe Lamp with visible filter (> 420nm)	355	100	Continuous flow reactor	This work
TiO_2 (P25)	Purged CO_2 in Water	300W Xe Lamp	~0.5	4	Liquid Phase Batch	[1]
TiO_2 (P25)	CO_2 gas with water drop	300W Xe Lamp	2.12	1	Gas Phase Batch	[2]
TiO_2 (P25)	Humified CO2	Solar simulator 1 Sun with a 425 nm cutoff filter	0.5	6	Gas Phase Batch	[3]
TiO_2	CO_2 gas with water drop	300W Xe Lamp	3.97	3	Gas Phase Batch	[4]
TiO_2	CO_2 gas with water drop	300W Xe Lamp	3.54	10	Gas Phase Batch	[5]
TiO_2	CO_2 gas	500W Hg lamp (>254 nm)	2.6	0.8	Gas Phase Batach	[6]
TiO_2	CO_2 gas with water drop	300W Xe Lamp	0.4	5	Gas phase batch	[7]
TiO_2	CO_2 gas with water drop	300W Xe Lamp	6.37	4	Gas phase batch	[8]
TiO_2	CO_2 gas with solution drop	300W Xe Lamp	10.2	<36	Gas phase batch	[9]
TiO_2	Purged CO_2 in Water	300W Xe Lamp	~0.25	2	liquid phase batch	[10]
TiO_2	Humified CO_2 (1000 ppm)	100W Xe Lamp (1.5 AM filter)	21.6 ppm/g h	1	Gas phase batch	[11]
ZnO	CO_2 gas with water drop	500W Xe Lamp with visible filter (> 420nm)	9	4	Gas Phase Batch	[12]
ZnO	CO_2 and H_2O vapor	350W Xe Lamp	0.64	1	Gas Phase Batch	[13]

ZnO	CO_2 gas with water drop	300W Xe Lamp with filter (320 nm < λ < 780 nm)	~1.16	4	Gas Phase Batch	[14]
$Cd_{1-x}S$	CO_2 gas (1000 ppm) with water drop	300W Xe Lamp	0.3	2	Gas Phase Batch	[15]
CdS_{1-x}	CO_2 gas (1000 ppm) with water drop	300W Xe Lamp	0.7	2	Gas Phase Batch	[15]
CdS	CO_2 and H_2O vapor	300W Xe Lamp with visible filter (> 420nm)	0.21	1	Gas Phase Batch	[16]
C_3N_4	CO_2 gas with water drop	500W Xe Lamp with visible filter (> 420nm)	9.4	4	Gas Phase Batch	[12]
C_3N_4	CO_2 and H_2O vapor	300W Xe Lamp (1.5 AM filter)	4.33	1	Gas Phase Batch	[17]
C_3N_4	CO_2 gas with water drop	300W Xe Lamp	17.675	4	Gas Phase Batch	[18]

[a]Photocatalysts for CO_2 photoreduction. [b]Production rate calculated based on the amount of total products formed during CO_2 reduction reaction, with values shown in parentheses. [d]Test time for photocatalytic durability including total cycle time. [e]Reaction system, either a static pressurized gas system or CO_2-purged water based liquid system.

Supplementary Table 2 | Performance comparison with state-of-the-art photocatalytic CO_2 reduction systems under various catalyst designs and reaction conditions.

Photocatalyst[a]	Reaction Medium	Light Source	Production Rate[b] (μmol/g•hr)	Stability[c] (hr)	Reaction system[d]	Ref.
TiO_2 (P25)	CO_2 and H_2O flow	300W Xe Lamp	686 (CO: 686)	360	Continuous flow reactor	This work
CuAu-DAs-TiO_2	CO_2 and H_2O	300W Xe Lamp	483.2 (C_2H_4: 568.8)	120	Intermittent flow system	[19]
Co_3O_4-TiO_2/CdS-CuO_x	CO_2 gas with water drop	300 W Xe lamp	126 (CO: 124 CH_4: 2.5)	20	Gas Phase Batch	[20]
GDY-Cu/ZnO	CO_2 gas with water drop	300W Xe Lamp	35.79 (CO: 2.12 CH_4: 33.67)	40	Gas Phase Batch	[21]
Pd_1-Bi_1 Bi_2O_3/TiO_2	CO_2 gas with water drop	300W Xe Lamp	212.5 (C_2H_6: 212.5)	50	Gas Phase Batch	[22]
Br-COF @ BiOCl	CO_2 gas from $NaHCO_3$+H_2SO_4	300W Xe Lamp with 320nm filter	27.4 (CO: 27.4)	20	Gas phase batch	[23]
$Cs_2AgBiBr_6$ @Co_3O_4	CO_2 gas with water drop	300W Xe Lamp with visible filter (> 420nm)	~200 (CO: ~200)	10	Gas phase batch	[24]
Co-doped CdS	CO_2 purged solution (H_2O+CH_3CN+TEA)	300W Xe Lamp	2322.8 (CO: ~2322.8)	20	Liquid phase batch	[25]
Cu_2O-Pt/SiC/IrO_x	CO_2 bubbled to solution (2 mM $FeCl_2$)	300W Xe Lamp with visible filter (> 420nm)	896.7 (HCOOH: 896.7)	40	Liquid phase batch	[26]

[a]Photocatalysts for CO_2 photoreduction. [b]Production rate calculated based on the amount of total products formed during CO_2 reduction reaction, with values shown in parentheses. [d]Test time for photocatalytic durability including total cycle time. [c]Reaction system, either a static pressurized gas system or CO_2-purged water based liquid system.

References

1. Kumar, S. *et al.* P25@CoAl layered double hydroxide heterojunction nanocomposites for CO 2 photocatalytic reduction. *Appl. Catal. B* **209**, 394–404 (2017).
2. Wang, L. *et al.* Anchored Cu(II) tetra(4-carboxylphenyl)porphyrin to P25 (TiO2) for efficient photocatalytic ability in CO2 reduction. *Appl. Catal. B* **239**, 599–608 (2018).
3. Hwang, H. M. *et al.* Phase-selective disordered anatase/ordered rutile interface system for visible-light-driven, metal-free CO2 reduction. *ACS Appl. Mater. Interfaces* **11**, 35693–35701 (2019).
4. Ye, M., Wang, X., Liu, E., Ye, J. & Wang, D. Boosting the photocatalytic activity of P25 for carbon dioxide reduction by using a surface-alkalinized titanium carbide MXene as cocatalyst. *ChemSusChem* **11**, 1606–1611 (2018).
5. Chen, Q. *et al.* Z-scheme Bi/AgBiS2/P25 for enhanced CO2 photoreduction to CH4 and CO with photo-themal synergy. *Appl. Surf. Sci.* **555**, 149648 (2021).
6. Xu, C. *et al.* Photothermal coupling factor achieving CO2 reduction based on palladium-nanoparticle-loaded TiO2. *ACS Catal.* **8**, 6582–6593 (2018).
7. Wang, Z. *et al.* Enhanced photocatalytic CO2 reduction over TiO2 using metalloporphyrin as the cocatalyst. *Catalysts* **10**, 654 (2020).
8. Jung, H. *et al.* Highly efficient and stable CO_2 reduction photocatalyst with a hierarchical structure of mesoporous TiO_2 on 3D graphene with few-layered MoS_2. *ACS Sustain. Chem. Eng.* **6**, 5718–5724 (2018).
9. Su, K. *et al.* In situ coating CsPbBr3 nanocrystals with graphdiyne to boost the activity and stability of photocatalytic CO2 reduction. *ACS Appl. Mater. Interfaces* **12**, 50464–50471 (2020).

10. Jiang, Z. *et al.* Living atomically dispersed Cu ultrathin TiO2 nanosheet CO2 reduction photocatalyst. *Adv. Sci. (Weinh.)* **6**, 1900289 (2019).

11. Lee, B.-H. *et al.* Electronic interaction between transition metal single-atoms and anatase TiO2 boosts CO2 photoreduction with H2O. *Energy Environ. Sci.* **15**, 601–609 (2022).

12. He, Y., Wang, Y., Zhang, L., Teng, B. & Fan, M. High-efficiency conversion of CO2 to fuel over ZnO/g-C3N4 photocatalyst. *Appl. Catal. B* **168–169**, 1–8 (2015).

13. Nie, N., Zhang, L., Fu, J., Cheng, B. & Yu, J. Self-assembled hierarchical direct Z-scheme g-C3N4/ZnO microspheres with enhanced photocatalytic CO2 reduction performance. *Appl. Surf. Sci.* **441**, 12–22 (2018).

14. Zhang, F., Li, Y.-H., Qi, M.-Y., Tang, Z.-R. & Xu, Y.-J. Boosting the activity and stability of Ag-Cu2O/ZnO nanorods for photocatalytic CO2 reduction. *Appl. Catal. B* **268**, 118380 (2020).

15. Cao, Y. *et al.* Modulating electron density of vacancy site by single Au atom for effective CO2 photoreduction. *Nat. Commun.* **12**, 1675 (2021).

16. Yu, J., Jin, J., Cheng, B. & Jaroniec, M. A noble metal-free reduced graphene oxide–CdS nanorod composite for the enhanced visible-light photocatalytic reduction of CO2 to solar fuel. *J. Mater. Chem. A Mater. Energy Sustain.* **2**, 3407 (2014).

17. Wang, J. *et al.* A single Cu-center containing enzyme-mimic enabling full photosynthesis under CO2 reduction. *ACS Nano* **14**, 8584–8593 (2020).

18. Ma, M. *et al.* Ultrahigh surface density of Co-N2C single-atom-sites for boosting photocatalytic CO2 reduction to methanol. *Appl. Catal. B* **300**, 120695 (2022).

19. Xie, Z. *et al.* Well-defined diatomic catalysis for photosynthesis of C2H4 from CO2. *Nat. Commun.* **15**, 2422 (2024).

20. *Hollow TiO2/CdS Z-Scheme Heterojunctions with Spatially Separated Cocatalysts for Highly Selective Photodriven CO2 Conversion*.
21. Han, Y. *et al.* Slow-light-driven photocatalytic CO2 reduction to CH4 mediated by photonic crystal Graphdiyne-Cu/ZnO Z-scheme system. *Chem. Eng. J.* **500**, 157636 (2024).
22. Chen, Q. *et al.* Enhancing local CO2 availability via amorphous Bi2O3 enable efficient and stable photocatalytic C2H6 production. *Chem. Eng. J.* **504**, 158854 (2025).
23. Wang, Y. *et al.* N-Bi covalently connected Z-scheme heterojunction by in situ anchoring BiOCl on triazine-based bromine-substituted covalent organic frameworks for the enhanced photocatalytic reduction of CO2 and Cr (VI). *Chem. Eng. J.* **505**, 159349 (2025).
24. Song, Y. *et al.* Boosted photocatalytic CO2 conversion of a Cs2AgBiBr6@Co3O4 composite with high activity and selectivity under low-concentration CO2 and natural sunlight. *Appl. Catal. B* **363**, 124816 (2025).
25. Xiong, R. *et al.* Dual electronic-spin engineering in cobalt-doped CdS: Photocatalytic pathway switching from competing H2 evolution to selective CO2 reduction. *Chem. Eng. J.* **520**, 166224 (2025).
26. Wang, Y. *et al.* Direct and indirect Z-scheme heterostructure-coupled photosystem enabling cooperation of CO2 reduction and H2O oxidation. *Nat. Commun.* **11**, 3043 (2020).
27. Jung, H. *et al.* Continuous-flow reactor with superior production rate and stability for CO_2 reduction using semiconductor photocatalysts. *Energy Environ. Sci.* **16**, 2869–2878 (2023).
28. Jung, H. *et al.* Continuous flow photoelectrochemical reactor with gas permeable photocathode: Enhanced photocurrent and partial current density for CO2 reduction. *Adv. Sci. (Weinh.)* **12**, e2411348 (2025).

29. Ye, Y.-X. *et al.* Visible-light driven efficient overall H2O2 production on modified graphitic carbon nitride under ambient conditions. *Appl. Catal. B* **285**, 119726 (2021).
